# Default Mode Hypoconnectivity Underlies a Sex-Related Autism Spectrum

**DOI:** 10.1016/j.bpsc.2016.04.006

**Published:** 2016-07

**Authors:** Rolf J.F. Ypma, Rachel L. Moseley, Rosemary J. Holt, Naresh Rughooputh, Dorothea L. Floris, Lindsay R. Chura, Michael D. Spencer, Simon Baron-Cohen, John Suckling, Edward T. Bullmore, Mikail Rubinov

**Affiliations:** a Brain Mapping Unit, Department of Psychiatry; b Autism Research Centre, Department of Psychiatry; c Hughes Hall; d Behavioural and Clinical Neuroscience Institute, Department of Experimental Psychology; e; Churchill College, University of Cambridge, Cambridge; f CLASS Clinic, Cambridge; g Cambridgeshire and Peterborough Foundation Trust, Cambridge; h Bournemouth University, Dorset; i ImmunoPsychiatry, Alternative Discovery & Development, GlaxoSmithKline, Stevenage, United Kingdom; j Janelia Research Campus, Howard Hughes Medical Institute, Ashburn, Virginia

**Keywords:** Autism, Connectomics, Default mode network, Extreme male brain theory, Functional connectivity, Neuroimaging

## Abstract

**Background:**

Females and males differ significantly in the prevalence and presentation of autism spectrum conditions. One theory of this effect postulates that autistic traits lie on a sex-related continuum in the general population, and autism represents the extreme male end of this spectrum. This theory predicts that any feature of autism in males should 1) be present in autistic females, 2) differentiate between the sexes in the typical population, and 3) correlate with autistic traits. We tested these three predictions for default mode network (DMN) hypoconnectivity during the resting state, one of the most robustly found neurobiological differences in autism.

**Methods:**

We analyzed a primary dataset of adolescents (*N* = 121, 12–18 years of age) containing a relatively large number of females and a replication multisite dataset including children, adolescents, and adults (*N* = 980, 6–58 years of age). We quantified the average connectivity between DMN regions and tested for group differences and correlation with behavioral performance using robust regression.

**Results:**

We found significant differences in DMN intraconnectivity between female controls and females with autism (*p =* .001 in the primary dataset; *p =* .009 in the replication dataset), and between female controls and male controls (*p =* .036 in the primary dataset; *p =* .002 in the replication dataset). We also found a significant correlation between DMN intraconnectivity and performance on a mentalizing task (*p =* .001) in the primary dataset.

**Conclusions:**

Collectively, these findings provide the first evidence for DMN hypoconnectivity as a behaviorally relevant neuroimaging phenotype of the sex-related spectrum of autistic traits, of which autism represents the extreme case.

The strikingly high male to female prevalence ratio is one of the most obvious and robust characteristics of autism spectrum conditions (ASCs) (1, 2, 3). While it is not known whether this bias reflects differential rates of diagnosis or genuine sex differences in prevalence (4, 5), the link between autism and the male sex is common in pervasive public stereotypes and originates with the first descriptions of these conditions. Asperger, having never encountered a female patient, informally described his eponymous syndrome as an “extreme variant of male intelligence” (6). A later formulation of this original observation noted population-level differences between the sexes in systemizing (i.e., a tendency to think mechanistically and logically, to perceive patterns and systems) and empathizing (i.e., the ability to identify and affectively share the emotional states of others), which are respectively stronger and weaker in patients with ASCs (7, 8). Males typically show an attenuated version of the same trend (i.e., greater systemizing and lower empathizing), and therefore these observations have led to the hypothesis that autistic traits exist on a continuum in the typical population [a prediction borne out by genetic studies (9)] and that ASCs represent an extreme form of the typical male brain (7, 8, 10, 11).

The neurobiological underpinnings of this framework have received little attention. Most neuroimaging studies have focused on identifying neurobiological features of autism, usually in predominantly male populations. The parsimonious prediction generated by the framework is that such neurobiological differences in ASCs would further reflect the “extreme” position of these individuals on the spectrum on which typical males and females differ. More specifically, a robust neurobiological feature of autism in males would 1) be similarly present in females with ASC, 2) show sex-specific differences in the typically developing population, and 3) correlate with autistic behavioral traits. However, these predictions have not been tested.

Rather than examining brain areas in isolation, much autism research has focused on functional connectivity patterns between brain regions (12). Functional connectivity is defined as the statistical association between pairs of brain regions, and may be inferred across a range of spatial and temporal scales, with a variety of measures. In the present article, we focus on the most common operationalization of this concept in human neuroimaging: the computation of functional magnetic resonance imaging (fMRI) connectivity using Pearson correlation (13). The default mode network (DMN) has been of particular interest in people with ASCs because of its putative role in mentalizing and social cognition (14, 15, 16, 17, 18). This network, a group of brain regions that reduce their activity during cognitive processing, fails to deactivate in people with ASCs (19, 20, 21). Altered functional connectivity between DMN regions at rest (22, 23, 24, 25, 26, 27, 28, 29) and during tasks (30) is among the most commonly reported functional connectivity findings in people with ASCs. Differences in functional connectivity within this network have been found across a range of methods, including independent component analysis (22, 24), region of interest or seed-based analyses (23, 26, 27, 28, 29), and graph-theoretical analyses (25, 30). In addition, these differences correlate with core ASC symptoms (22, 23, 27, 28) and constitute an endophenotype (21, 30)—a genetically mediated biomarker (31) that distinguishes biological relatives of people with ASC from other members of the population. However, the current literature on sex differences in DMN connectivity is sparse and inconsistent (32, 33, 34). Alteration of DMN connectivity therefore represents a natural target for investigation of the hypothesized sex-related spectrum of autistic traits.

We leveraged a primary dataset with a relatively large number of females with ASC, female siblings of individuals with ASC, and a replication multisite dataset to robustly test the predictions made by this framework. All participants were scanned during resting state—that is, a condition of lying still and quietly, unengaged in cognitive tasks. We specifically tested whether weaker functional connectivity between regions of the DMN 1) is a feature and endophenotype for autism in females, as has previously been shown in males with autism, 2) is present in males relative to females in the typically developing population, and 3) correlates with decreased mentalizing ability, typically affected in autism. We also tested the specificity of DMN hypoconnectivity by leveraging a positive control dataset of participants with a distinct psychiatric condition, major depressive disorder.

## Methods and Materials

### Primary Dataset: The Cambridge Family Study of Autism

The Cambridge Family Study of Autism (CFSA) comprises resting-state and task scans from control females (*n* = 20), control males (*n* = 20), females with ASC (*n* = 16), males with ASC (*n* = 35), and nonaffected female (*n* = 30) and male (*n* = 13) siblings of subjects with ASC (21, 30, 35, 36, 37, 38, 39). We used only the resting state scans from this sample for analysis. All participants were 12 to 18 years of age, had no history of psychotropic drug use, and had no other documented psychiatric conditions. Diagnostic status of people with ASCs was confirmed with the Autism Diagnostic Observation Schedule–Generic and Autism Diagnostic Interview-Revised assessments, which are gold standard tools in autism diagnosis (40, 41) (see Supplement for full demographic details of all participants).

### Replication Dataset: ABIDE

To test the reproducibility of findings from the CFSA data, we analyzed resting-state scans from 408 males with ASC, 428 control males, 55 females with ASC, and 89 control females obtained from the Autism Brain Imaging Data Exchange (ABIDE) (42). These data were collected from 15 imaging sites, and participants spanned a wide age range (6–58 years of age). However, 456 (47%) participants were in the same age range (12–18 years of age) as the CFSA participants. This replication dataset offers a considerable increase in statistical power at the expense of a more heterogeneous population. We discuss our control for this heterogeneity below.

### Positive Control Dataset: Magnetic Resonance Imaging for Myocardial Perfusion Assessment in Coronary Artery Disease Trial (MR-IMPACT) Study of Depression

To test the specificity of our findings, we analyzed a positive control dataset of a distinct psychiatric disorder, major depression. We obtained data from the MR-IMPACT study of depression, which comprised resting-state scans from adolescent male (*n* = 6) and female (*n* = 18) controls, and adolescent male (*n* = 17) and female (*n* = 46) patients with moderate to severe major depressive disorder (43) but otherwise typical development (44).

### Preprocessing

fMRI scanning parameters for the primary and replication dataset are provided in the Supplement. A preprocessing pipeline using AFNI (45) and FMRIB Software Library (46) was applied to all scans. The pipeline included removal of the first five scans of each functional echo planar imaging series, skull-stripping, brain segmentation, nonlinear registration to Montreal Neurological Institute space, and coregistration of anatomic images to realigned and slice-time corrected functional scans. Motion parameters and mean signal from trimmed binary masks (partial volume estimates >0.99) of cerebrospinal fluid and white matter, their derivatives and quadratic terms, were regressed out as confounds, resulting in a total of 32 regressors (47, 48); we did not perform global signal regression (49). Each participant’s time series were despiked, band-pass filtered in the range of 0.01 to 0.1 Hz, denoised by removal of the 32 regressors (i.e., band-pass filtered in the same range), and smoothed with an 8-mm full width at half maximum Gaussian kernel, all using the AFNI 3dBandpass command (https://afni.nimh.nih.gov/pub/dist/doc/program_help/3dBandpass.html). Movement is an issue of high concern in analyses of functional connectivity (50, 51, 52, 53), and we provide details of our pipeline and an analysis of the effect of motion on our results in the Supplement.

The DMN was defined as 58 8-mm-radius spherical regions of interest derived from a meta-analysis of fMRI studies (Supplement) (54). To remove weak and spurious correlations, we analyzed binary networks obtained by thresholding the matrices and preserving only the strongest 20% of connection weights for each participant.

### Statistical Analysis

We computed functional DMN intraconnectivity as the density of all binary intra-DMN edges minus a constant number of such edges expected in a random network (0.2 for a 20% density; see Supplement for additional discussion). We defined functional connectivity using Pearson’s correlation and subtracted the constant to increase interpretability and decrease dependence of the measure on the chosen binarizing threshold. In the primary dataset, we tested for a difference between control females and 1) females with ASC, 2) sisters of subjects with ASC, 3) control males, and 4) males with ASC, and between control males and males with ASC, using multiple regression controlling for effects of age and IQ. We conducted the same tests in the replication dataset (with the exception of the sibling contrast) but included an additional regressor of study site (thereby correcting for age, IQ, and study site). The residuals of the test on the replication dataset failed a Shapiro-Wilk test for normality (*p =* .03), so we used robust regression for all analyses in this study, although results were similar with standard least squares regression. Robust regression, in comparison to standard regression, is less affected by violations of normality and by the potential presence of outliers (55).

We quantified the final effect sizes by pooling all available data from the primary and replication datasets and performing a multiple regression analysis, correcting for age, IQ, and study site. We quantified differences in connectivity between groups as a percentage change of mean DMN intraconnectivity relative to a baseline of control males (Supplement). We tested the specificity of observed effects by repeating the multiple regression analysis on all data including the positive control dataset, specifically testing for an effect of depression diagnosis. We repeated this test separately for both sexes. Finally, we explicitly investigated the effect of age, repeating the analysis on the pooled data of the primary and replication datasets stratified by age group: children (6–11 years of age), adolescents (12–18 years of age), and adults (>18 years of age).

### Robustness Analyses

We conducted a number of robustness analyses, including the additional preprocessing step of scrubbing, exclusion of high-moving subjects, regressing out motion parameters, using a threshold-independent quantification of intranetwork connectivity, and excluding three sites in the ABIDE dataset associated with previous studies of DMN connectivity (to exclude a possible circular argument). We also further explored the impact of motion. See the Supplement for full details on these analyses.

### Behavioral Analysis

Data were collected for all CFSA participants on performance on the “Reading the Mind in the Eyes” mentalizing task (38, 56). This task, performed during fMRI recording, is a popular test of mentalizing and emotion recognition: presented with just a pair of eyes, participants were required to choose one of two words to describe the expression of the eyes and the congruent mental state. Although we do not have direct measures of systemizing or empathizing, this task is related to mentalizing and the empathizing construct. In a control, nonmentalizing condition, participants simply judged whether the eyes belonged to a male or female. Previously, we found performance on this mentalizing task to be related to diagnosis; subjects with autism performed worse than controls (38). We examined whether DMN intraconnectivity correlated with the percentage of incorrect responses in the mentalizing and the control condition beyond this diagnosis effect by regressing out the effects of age, IQ, and the six groups (i.e., ASC, sibling, and control groups split by sex). We also performed this analysis separately for the two sexes and three groups.

## Results

**Figure 1:**
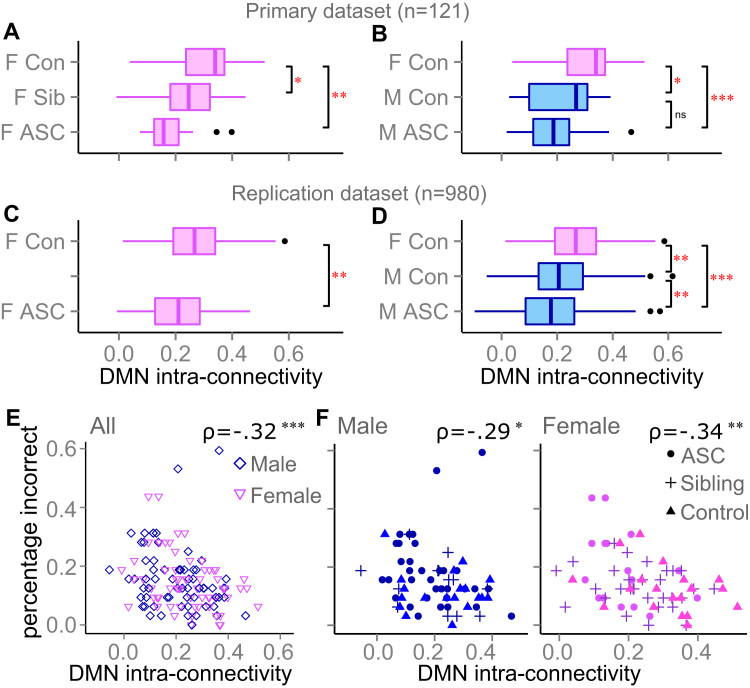
Predictions for default mode network (DMN) connectivity in a sex-related autistic trait spectrum. Group differences in DMN intraconnectivity for **(A)** 66 and **(B)** 75 participants from the primary dataset (20 female controls present twice) and **(C)** 144 and **(D)** 925 participants from the replication dataset (89 female controls present twice). **(E, F)** Relationships between DMN intraconnectivity and performance on a mentalizing task, Spearman rho is given. All data are shown in **(E)** and split by sex in **(F)**. Box plots give quartiles and asterisks reflect significant differences (*p < .05, **p < .01, and ***p < .001). ASC, autism spectrum condition; Con, controls; F, females; M, males; Sib, siblings.

DMN hypoconnectivity was previously shown to characterize autism in male-only or heavily male-biased studies (22, 23, 24, 25, 26, 27, 28, 29, 30) and to appear as an endophenotype in male siblings (30). Here, controlling for heterogeneity in age and IQ, we found that DMN hypoconnectivity is likewise robustly present in females with ASC (primary dataset *p* = .001; replication dataset *p* = .009; Figure 1A, C). We further found that it represents an endophenotype, with unaffected female siblings of individuals with ASC placed between typically developing and autistic participants, and having significantly lower connectivity than the former (*p* = .035). The endophenotype analysis of the females complements our previous report of an endophenotype for the male-only subset of this dataset in a previous study (30). In addition, consistent with the hypothesized difference in autistic traits between typical males and females, DMN intraconnectivity was lower in control males than control females (primary dataset *p* = .036; replication dataset *p* = .002; Figure 1B, D).

**Figure 2:**
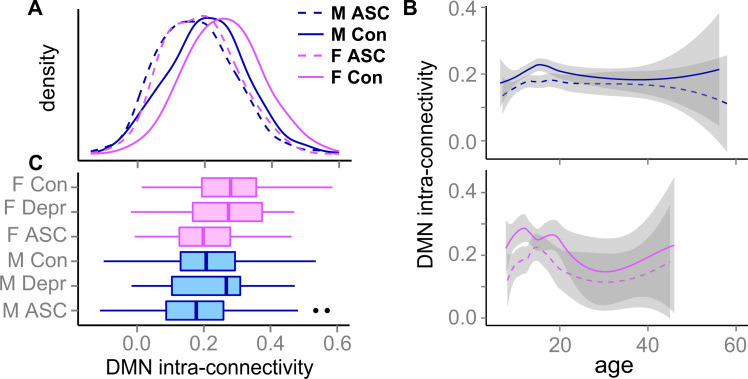
Default mode network (DMN) intraconnectivity distributions derived from pooling the primary and replication datasets. The effects of age, IQ, and site have been regressed out. **(A)** The distribution of DMN intraconnectivity for the 4 groups (top) and the 4 groups and positive control participants with major depressive disorder (bottom). The latter category does not differ from the control subjects. The panel shows both a clear difference between the mean values of the groups and large within-group heterogeneities. **(B)** The effect of age on these values. The lines for females are more volatile because of lower numbers, especially for adult ages. ASC, autism spectrum condition; Con, control; Depr, depression; F, female; M, male.

We quantified the effect sizes for the four groups by pooling the primary and replication datasets (Figure 2A). The mean connectivity for control females was 27% higher than the mean value for control males, while the mean for males with ASC was 16% lower. Females with ASC were intermediate between males with ASC and control males, with a mean 9% lower than the latter (not statistically significantly different from either group, *p* > .1).

Our pooled data covered a substantial age range (6.5–58 years of age). Although DMN intraconnectivity appears variable across the lifespan (Figure 2B), the contrasts we identified were present to some extent in all three age groups. We replicated all three testable comparisons, control females versus females with ASC, control females versus control males, and control males versus males with ASC for children (effect size [*p* value]: 0.08 [.009], 0.06 [.01], and 0.04 [.02]) and adolescents (0.06 [.02], 0.05 [.002], and 0.03 [.004]). For adults, effect sizes were reduced and differences were trend level or nonsignificant (0.08 [.1], 0.02 [.5], and 0.03 [.1]). See the Supplement for details of this analysis. We could not evaluate the endophenotype effect in the replication dataset because it did not contain siblings.

We found no significant default mode connectivity effect in a positive control dataset of participants (*n* = 63) diagnosed with a distinct psychiatric condition, major depressive disorder (*p* > .1; Figure 2C). The effect remained absent when allowing for a sex by diagnosis interaction (see Supplement).

Behavioral data are available from the CFSA sample only. The observed differences in DMN connectivity were significantly associated with performance on the “Reading the Mind in the Eyes” task (56), a task known to reveal mentalizing impairments in people with autism (Figure 1E, F). A higher percentage of errors on this task was associated with lower DMN intraconnectivity in the whole sample of males and females with ASC, siblings, and controls (*p* = .001) beyond the effects of diagnosis, age, and IQ. The same effect was separately found in the female (*p* = .034) and male (*p* = .016) groups. A negative effect was also found when analyzing each of the three groups separately (i.e., ASC, siblings, and controls), but only significantly so for the ASC group (*p* = .008; see the Supplement for the full results). In addition, there was no relationship between DMN intraconnectivity and percentage errors in a control task of gender judgment (*p* > .1), and performance on neither task correlated with movement (see Supplement).

## Discussion

To our knowledge, this is the first investigation of functional connectivity in the DMN as a neurobiological correlate of the sex-related spectrum of autistic traits. We used two independently acquired datasets to test three specific predictions. First, we showed a robust and specific reduction in DMN intraconnectivity in females with ASC and in unaffected female siblings of subjects with ASC, replicating previous results in males (30). Second, we found that control females had an increased DMN intraconnectivity compared to control males, and that people with ASC tended to have lower intraconnectivity still. Third, and in line with these findings, we found that DMN intraconnectivity correlated with performance in a behaviorally relevant mentalizing task that typically reveals deficits in autism. These findings bring together two strands of research in the autism literature, suggesting that abnormal DMN connectivity may underlie the spectrum of autistic traits that extends into the general population. Reduced DMN intraconnectivity is consistently found across males and females on the spectrum, and indeed differs in typically developing males and females, and we suggest that it may be highly relevant to the autistic phenotype and autistic traits that appear to a greater extent in males.

The idea that ASCs resemble an exaggerated manifestation of typical sexual dimorphism was originally linked to the expression of systemizing and empathizing (7, 8), psychological processes linked respectively to strengths and weaknesses in autism. To a lesser extent than people with autism, typically developed males also tend to show strengths in the former and weaknesses in the latter; consequently, people with autism were said to show a form of the “extreme male brain” (7, 8). This theory was extended in later years after a tentative relationship was found between empathizing, systemizing, autistic traits, and prenatal androgen exposure (10, 11), which is believed to permanently modify brain structure (57, 58). Although we cannot comment on this aspect of the male brain hypothesis, we add to this original theory by revealing that the most robust difference in functional connectivity in ASC is expressed on the same sex-related spectrum.

Many studies suggest that ASC in females is distinct at the level of brain and behavior from ASC in males (4, 59, 60, 61, 62, 63, 64, 65, 66). Research continues to search for differences in genetics and for protective features that might set apart females with ASC (4, 67). There are nevertheless some brain and behavioral commonalities between males and females with ASC (4, 59, 60), and our study is to our knowledge the first functional connectivity investigation to report that reduced DMN intraconnectivity is shared across the sexes, with both males and females with ASC down the more “male” end of the spectrum (Figure 2A). This finding, consistent with behavioral results (68), indicates that DMN connectivity may underlie some of the shared symptomatology of autism in males and females, and fits well with the putative role of DMN in mentalizing and social cognition (14, 15, 16, 17, 18), known to be impaired in both males and females with ASC. Further evidence of this role was given by the correlation we observed between DMN intraconnectivity and performance on the mentalizing task, which relies on some of the same cognitive mechanisms as empathy (7), and lack of correlation with performance in a condition unrelated to mentalizing (gender judgments). It remains unknown whether these hypotheses apply to other aspects of brain connectivity; results from a recent study are consistent with the DMN being unique in this regard (69).

The effects of age on functional connectivity in ASC has been a topic of recent interest (70). DMN intraconnectivity has been studied in children (23, 71), adolescents (22, 25, 28, 30), and adults (27) with ASC, and in wide-ranging groups spanning late adolescence to adulthood (24, 26, 29). On the whole, these lean toward hypoconnectivity, with the exception of three studies that, upon greater scrutiny, report hyperconnectivity between some individual nodes of the DMN (23, 25, 71) in contrast to the more expansive approach we took here. This may explain why, when studying the DMN as a larger whole, we saw reductions in DMN intraconnectivity that were common across age groups in ASC, appearing both in a tight age-matched group (12–18 years of age) and a larger dataset with wide age range (6–58 years of age). Additional analyses on the latter revealed the effects to be significant for children (6–11 years of age) and adolescents (12–18 years of age) but only at trend level for adults (19–58 years of age). This seems consistent with the view that ASCs are developmental conditions in which neurobiological differences may be at their most apparent in early life (72, 73). There has been the suggestion of general hyperconnectivity in early life in people with ASC (70), and this was indeed seen between some nodes of the DMN (23, 71) but does not appear to be the case for DMN as a whole. Other studies, finding smaller and absent effects in adulthood and adolescence, respectively, suggest that DMN connectivity develops on a markedly different trajectory in ASC (74, 75); greater than average variability in the rate of development in people with ASC could explain the null findings from these studies. The current study used a cross-sectional sample, and a longitudinal analysis in future research may help to clarify age-related changes in the DMN. Further research should also clarify whether effects of sex, as defined in the extreme male brain theory, are modulated by age.

It is important to note that our results apply to group differences and tendencies in large populations, and therefore may not fully explain individual differences. For example, even though people with ASC tend to fall on the (extreme) male end of the distribution (76), this is not true of every individual. This is well depicted in the distribution of the data in Figure 2: the different groups show clearly different profiles, all characterized by a large amount of within-group heterogeneity. In line with current views on insufficient emphasis on effect sizes (77, 78, 79), it is important to realize that heterogeneity can mask considerable effects. Indeed, we found an increase of 27% (respectively a decrease of 16%) in DMN connectivity for control females (respectively for ASD males) relative to control male participants. These findings suggest that DMN intraconnectivity represents an important risk factor in a multifactorial interplay underpinning the biological presentation of autism.

Motion can have a profound effect on estimates of functional connectivity (50, 51, 52, 53). Nontrivial patterns of distance-dependent alterations of functional connectivity have been shown to be the result of motion artifacts, and many preprocessing strategies, such as the one used in this study, have been used to correct for these. Motion is particularly problematic for studies of autism because participants with ASCs tend to move more in the scanner. However, our results were robust against a range of methods aimed to reduce motion artifacts, including the additional step of scrubbing (see Supplement). It is noteworthy, however, that a correlation with motion remained after these preprocessing steps. This remaining correlation is consistent with recent findings from Zeng *et al*. (80). These authors presented evidence that DMN hypoconnectivity is a stable biological trait that predisposes to movement rather than an artifact caused by scanner movement; they found that individuals with lower DMN connectivity tend to move more. We likewise found that DMN intraconnectivity correlated with motion in repeat scans for the same subjects, even after removing the effect of motion in the current scan (see Supplement). Importantly, we also found a strong relationship between DMN intraconnectivity and performance on a mentalizing task, providing additional, albeit indirect, evidence for the claims of Zeng *et al*. that the correlation may represent biological rather than artifactual effects. Future research should evaluate this hypothesis in more detail, investigating to which groups and under which circumstances it applies.

A notable feature of our study is the inclusion of a positive control group of patients with a distinct psychiatric disorder, major depression. This inclusion differentiates our work from the majority of neuroimaging autism literature, which does not include positive control subjects. In contrast to autism, there seems to be, on balance, greater evidence for DMN hyperconnectivity, rather than hypoconnectivity, in adults with major depression (81, 82). Another study, reporting more complex patterns of hyper- and hypoconnectivity within and between DMN and other brain regions, suggests that developmental changes with age may impact findings (83). While our adolescent depression data set was comparatively small, the absence of DMN hypoconnectivity in these data, at least, represents some evidence for specificity of our studied connectivity measure. More generally, the inclusion of positive controls in future studies represents an important goal toward more clinically relevant conclusions, and constitutes an important step towards translation of this and other neuroimaging phenotypes in ASC (84, 85).

In summary, our analyses suggest that the DMN shows a robust, heritable, specific, and behaviorally relevant reduction across the autism spectrum. The analyses simultaneously reconcile two distinct strands of autism research—the extreme male brain theory of autism and default mode connectivity in autism—into a convergent and unified picture of biological abnormalities in autism.

## Footnotes

Appendix A Supplementary material cited in this article is available online at doi:10.1016/j.bpsc.2016.04.006.
