# Default Mode Hypoconnectivity Underlies a Sex-Related Autism Spectrum

**DOI:** 10.1016/j.bpsc.2016.08.004

**Published:** 2016-11

Erratum to: “Default Mode Hypoconnectivity Underlies a Sex-Related Autism Spectrum” by Ypma *et al*. (*Biol Psychiatry Cogn Neurosci Neuroimaging*; 2016, 1(4):364–371; http://dx.doi.org/10.1016/j.bpsc.2016.04.006).

A copyediting error was inadvertently introduced into the article on page 365. Specifically, the subheading that currently reads:

Positive Control Dataset: Magnetic Resonance Imaging for Myocardial Perfusion Assessment in Coronary Artery Disease Trial (MR-IMPACT) Study of Depression

should instead read:

Positive Control Dataset: Magnetic Resonance-Improving Mood with Psychoanalytic and Cognitive Therapies (MR-IMPACT) Study of Depression.

