# Supplementary material for: Default Mode Hypoconnectivity Underlies a Sex-Related Autism Spectrum
Source: Biol Psychiatry Cogn Neurosci Neuroimaging. 2016 Jul;1(4):364–71. doi: 10.1016/j.bpsc.2016.04.006 (PMC4936761; doi:10.1016/j.bpsc.2016.04.006)
Supplement: Supplementary file 1 — Supplementary material [file mmc1.pdf]

Default Mode Hypoconnectivity Underlies a Sex-Related Autism Spectrum

Supplemental Information

Participant Details 2

Primary Dataset: Cambridge Family Study of Autism 2
Replication Dataset: ABIDE 4
Positive Control Dataset: MR-Impact Adolescent Depression 5

Scanning Parameters 6

Primary Dataset 6
Replication Dataset 7
Positive-Control Dataset 7

Control for Head Motion Artifacts 7

Primary Dataset 8
Replication Dataset 9
Correlation Between DMN Connectivity and Motion 10

Parcellation of the Default Mode Network 12

Computation of the DMN Connectivity Metric 12

Computation of Difference in Connectivity Between Groups 13

Robustness Analyses 13

Exclusion of High-Motion Participants 13
Additional Preprocessing: Scrubbing 14
Threshold-Free Measure of Intra-DMN Connectivity 14
Regressing Out Motion 14
Excluding Studies Previously Reporting DMN Differences 15

Different Age Groups 16

Positive Control: Depression 18

Further Analysis of Behavioral Data 20

Supplemental References 23

Participant Details

Primary Dataset: Cambridge Family Study of Autism

For our first question, whether default mode network (DMN) connectivity constitutes a marker of autism in females, we included all of the medication-free adolescent girls diagnosed with an ASC (*n* = 16), typically-developing adolescent girls (*n* = 20), and unaffected female siblings (*n* = 30, 25 unrelated to ASC girls in our sample) whose data was collected as part of the Cambridge Family Study of Autism (CFSA) (1–7). All of the data and measures analyzed in this study were collected in these previous studies, which also confirmed the diagnostic status of the ASC group with gold-standard diagnostic tools (8; 9). Siblings and controls both scored below the cut-off that differentiates them from people with ASC on the Autism-Spectrum Quotient (AQ) (10) and the Social Communication Questionnaire (SCQ) (11). Ethical approval for this study was granted by the Cambridgeshire 1 Research Ethics Committee. Demographic details are displayed in Table S1.

Table S1. Demographic information for the female groups and *p* values reflecting matching between them.

	**Females with ASC (*n* = 16)**	**Female Siblings (*n* = 30)**	**Female Controls (*n* = 20)**	**Matching (*F*)**
Age	14.5 (2.0) [6.5]	14.6 (2.2) [6.9]	14.8 (1.7) [6.1]	$p = .851$
Full-scale IQ	97.6 (10.7) [45]	112 (9.6) [45]	110.7 (10.9) [43]	$p < .001$
Verbal IQ	96.2 (11.3) [40]	112.5 (13.4) [55]	110.5 (12.7) [45]	$p < .001$
Performance IQ	99.8 (16) [66]	109.7 (9.3) [40]	108.6 (8.8) [36]	$p = .017$
Autism-Spectrum Quotient	32.6 (15) [28]	7.5 (4.7) [24]	8.1 (4.6) [17]	$p < .001$
Social Communication Questionnaire	20.3 (7.3) [25]	1.07 (1.6) [7]	1.75 (2.3) [8]	$p < .001$

Numbers show the mean (standard deviation) [range].

We previously compared males with ASC, male siblings and controls in this dataset (6). In that study, we analyzed a subset of male participants, aiming to maximally match

participant demographics. Here, we used a regression strategy to control for heterogeneity, and so were able to include all 20 typically-developing control males and 35 males with ASC to answer our second question on the presence of DMN hypoconnectivity in the typical population. Demographics and matching with control females are displayed in Table S2.

Table S2. Demographic information for males with and without ASC and female controls, and *p* statistics reflecting matching between groups.

	**Female Controls (*n* = 20)**	**Male Controls (*n* = 20)**	**Males with ASC (*n* = 35)**	**Matching (*F*)**
Age	14.8 (1.7) [6.1]	15.3 (1.6) [5.3]	14.5 (1.7) [6.5]	$p = .65$
Full-scale IQ	110.7 (10.9) [43]	114 (11.4) [48]	108 (16.1) [65]	$p = .68$
Autism-Spectrum Quotient	8.1 (4.6) [17]	10.1 (6.4) [22]	39 (6.4) [28]	$p = .000$
Social Communication Questionnaire	1.75 (2.3) [8]	2.58 (2.3) [8]	25.3 (6.1) [26]	$p = .000$

Behavioral (mentalizing) and fMRI data was additionally collected for male siblings of people with autism (characteristics in Table S3). These were not relevant for the first two questions, but were included to assess the third question relating DMN intra-connectivity to task performance. This correlation remained significant in the absence of male siblings.

Table S3. Demographic information for male siblings of people with ASC, from the primary dataset (CFSA).

	**Male Siblings (*n* = 13)**
Age	15 (2.1) [6.6]
Full-scale IQ	113.5 (11) [32]
Verbal IQ	111.8 (12) [37]
Performance IQ	111.8 (11) [32]
Autism-Spectrum Quotient	11.3 (6) [23]
Social Communication Questionnaire	2.69 (2.9) [9]

Replication Dataset: ABIDE

We obtained data and demographic information from the Autism Brain Imaging Data Exchange (ABIDE: http://fcon_1000.projects.nitrc.org/indi/abide/) (12). We extracted participant age, available IQ measurements (provided as full-scale IQ, verbal IQ and/or performance IQ) and diagnosis. As the full three IQ scores were only available for a small set of participants, we took the average of the available scores as our measure of IQ. We then discarded all subjects for which either age, IQ or diagnosis was not available. We further discarded all studies for which the number of participants was less than 20, leaving 980 participants across 15 studies. We divided these up into the four relevant groups, which led to a sample of 89 control females, 428 control males and 55 females and 408 males with ASC. See Table S4 for the demographic information of each group, and Table S5 for a breakdown per study site.

Table S4. Demographic information for the participants used in the large-scale replication analysis, alongside matching (*F* statistics) between them.

	**Males with ASC (*n* = 408)**	**Females with ASC (*n* = 55)**	**Male Controls (*n* = 428)**	**Female Controls (*n* = 89)**	**Matching *(F)***
Age	16.3 (7.5) [51]	15.1 (7.3) [36]	16.9 (7.3) [49]	15 (6.2) [38]	$p = .073$
IQ	104.9 (16.4) [107]	104 (14.3) [53]	111 (11.5) [72]	108.9 (12.4) [57]	$p < .001$

Table S5. Number of ABIDE participants included from each institution.

Center	ASC	Controls	Total Number of Participants	Publications
University of California, LA (UCLA)	47 males<br>6 females	39 males<br>5 females	97	(13; 14)
University of Michigan (UM)	50 males<br>8 females	56 males<br>18 females	132	(15–17)
NYU Lagone Medical Center (NYU)	66 males<br>10 females	75 males<br>24 females	175	(18)
Yale Child Study Center (Yale)	19 males<br>8 females	18 males<br>8 females	53	-
University of Utah School of Medicine (USM)	56 males	42 males	98	(19; 20)
Stanford University (Stanford)	14 males<br>4 females	14 males<br>3 females	35	(21; 22)
Trinity Centre for Health Sciences (Trinity)	23 males	24 males	47	
San Diego State University (SDSU)	11 males<br>1 female	15 males<br>6 females	33	
University of Pittsburgh School of Medicine (Pitt)	26 males<br>4 females	23 males<br>3 females	56	
Olin Neuropsychiatry Research Center, Institute of Living at Hartford Hospital (Olin)	15 males<br>2 females	14 males<br>2 females	33	(23)
Oregon Health and Science University (OHSU)	11 males	12 males	23	
Ludwig Maximilians University Munich (MaxMun)	18 males<br>3 females	29 males<br>3 females	53	
University of Leuven	24 males<br>3 females	29 males<br>4 females	60	
Kennedy Krieger Institute (KKI)	17 males<br>4 females	23 males<br>9 females	53	
California Institute of Technology (Caltech)	11 males<br>2 females	15 males<br>4 females	32	(24)

Positive Control Dataset: MR-IMPACT Adolescent Depression

The positive control dataset contained participants from a Cambridge study on depression (25). Males and females met diagnostic criteria for major depressive disorder as defined by

DSM-IV (26). Patients with chronic and/or recurrent depressive episodes were included in the sample, as were those who were currently taking SSRI medication, but patients with drug/alcohol dependence, a learning disability, structural abnormalities of screening MRI scans, and/or importantly an ASC, were excluded from taking part. IQ data was not collected for all participants in the depressed groups. Ethical approval for this study was obtained from Cambridgeshire 2 Research Ethics Committee.

Table S6. Demographic information from participants recruited as part of the MR-IMPACT adolescent depression study (25).

	**Control Males (*n* = 6)**	**Control Females (*n* = 18)**	**Depressed Males (*n* = 17)**	**Depressed Females (*n* = 46)**
Age	15.7 (1.1) [3]	15.8 (1.7) [5]	15.6 (1.5) [5]	15.5 (1) [4]
IQ	101.7 (7) [20]	103.8 (12.3) [45]	-	-

Scanning Parameters

Primary Dataset

Functional and structural MRI scans for the participants in the first part of our analysis were acquired on a Siemens Tim Trio 3-T system (Siemens Healthcare, Erlangen, Germany) at the MRC Cognition and Brain Sciences Unit in Cambridge, UK. In a sequence lasting four minutes and 32 seconds, MPRAGE structural images were acquired with the following parameters: repetition time (TR) = 2250 ms, echo time (TE) = 2.98 ms, inversion time (TI) = 900 ms, flip angle 9º, voxel size 1 × 1 × 1 mm. Echoplanar images (EPI) during the functional tasks were acquired in a descending interleaved pattern with the following parameters: TR = 2000 ms, TE = 30 ms, flip angle 78º ,voxel size 3 × 3 × 3 mm, field of view = 192 × 192 mm, 64 × 64 acquisition matrix. Thirty-two slices were acquired with a slice thickness of 3 mm and an inter-slice distance of 0.75 mm.

Replication Dataset

Full details of individual site acquisitions are provided in the original studies (12).

Positive-Control Dataset

Functional and structural scans were acquired on a 3.0 Tesla Magnetom Trio Tim scanner (Siemens, Surrey, England) at the Wolfson Brain Imaging Centre, University of Cambridge, UK. High-resolution T1-weighted sequences were acquired in the sagittal plane using a three-dimensional magnetically prepared rapid acquisition gradient echo sequence (3D-MPRAGE; 176 slices of 1 mm thickness, TE = 2.98 ms, TR = 2.30 s, inversion time = 900 ms, flip angle = 9°, field of view = 240 × 256 mm, voxel size = 1 × 1 × 1 mm, series = interleaved). EPI depicting blood oxygen level-dependent (BOLD) contrast at a sampling time of 2.0 seconds were acquired during rest. MR data covering the whole brain were acquired using echo planar T2* weighted imaging. Thirty-two slices of data 3 mm in thickness parallel to the anterior-posterior commissure comprise each volume of data collected (TE = 30 ms, TR = 2 s, flip angle = 78°, and interleaved series). The field of view and voxel size were 192 × 120 mm and 3 × 3 × 3 mm, respectively.

Control for Head Motion Artifacts

Head motion during acquisition can create artifacts of dysconnectivity in functional imaging datasets (27–30). We conducted several quality control checks to examine the impact of motion on our functional connectivity (FC) estimates.

Firstly, we statistically compared motion parameters between groups by extracting six location parameters from the scans of each participant for each slice during the scan time-series. A measure of mean motion for each participant over the scan was derived by averaging the mean framewise displacement (FD) (31; 32): the sum of the absolute values of

the derivatives of the translational and rotational realignment estimates (after converting rotational estimates to displacement at 50 mm radius).

Secondly, for each pair of nodes, we computed the correlation between FC and mean and maximum framewise displacement. Successful reduction of movement artifacts would be reflected in a lack of correlation between FC and maximum framewise displacement, whilst movement distortion gives rise to higher correlations for short-distance node pairs and lower correlations for long-distance node pairs (33; 34). To assess the magnitude of the correlations, we performed the same computations after permuting FD values for the participants 100 times, thus generating the distribution of values to be expected when no relationship between motion and FC exists. We then tested, for both mean and maximum FD, if the overall mean correlation or the distance dependence is significantly different from those observed in the random permutations. The latter was tested by fitting a straight line through all correlation values and comparing the slope of this line to the slopes for each of the permutations.

Primary Dataset

We first tested the effect of motion on our data from the CFSA. Participants did not show gross movements in visual inspection of scans, and statistical analysis also showed no significant differences between the three female groups in mean motion (p = .126) or maximum motion (p = .264), and no differences in the other contrast of interest between control males and females for mean motion (p = .422) or maximum motion (p = .552). Figure S1 shows the moving average of the correlations between edge weights and motion as a function of Euclidean distance between nodes. The correlation between FC and mean FD, and between FC and maximum FD, was close to zero and showed little distance dependence. Both the slope and the magnitude of the correlation were not significantly different from the distribution obtained under the null hypothesis of no relation between FD and FC (p > .1).

These results indicate that our preprocessing pipeline was sufficient to remove global artifacts induced by motion from our connectivity estimates.

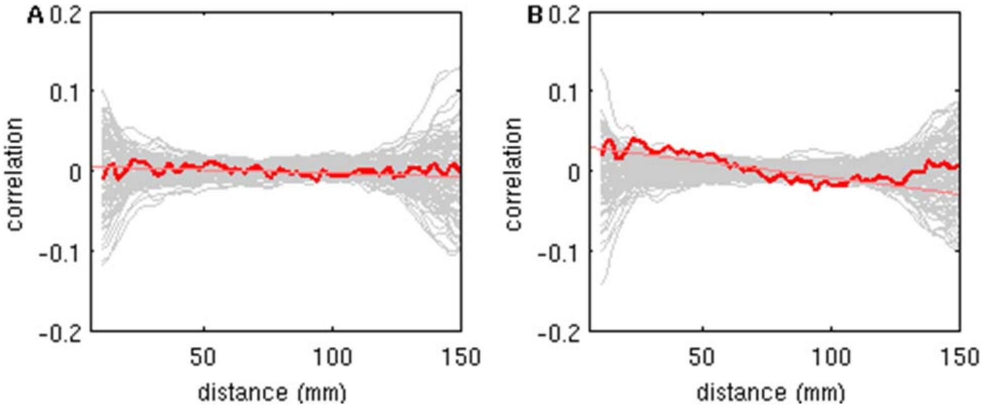

Figure S1. Moving average of correlation between maximum framewise displacement (**A**) or mean framewise displacement (**B**) and functional connectivity against distance between nodes, for the primary dataset. The bold red lines reflect values from actual data, whilst straight red lines are fitted linear functions: gray lines were obtained by permuting movement values for participants.

We also examined the relationship between movement parameters and DMN intra-connectivity specifically, with all five subject groups included (n = 121). DMN intra-connectivity correlated with mean motion ($r = -.290$, $p = .001$) and maximum motion ($r = -.319$, $p < .001$). These results together indicate that DMN is more related to motion than overall functional connectivity patterns.

Replication Dataset

We repeated these steps for the independent data of 980 participants. Both the mean correlation with mean FD [maximum FD] (-1.6×10^{-4} [-4.0×10^{-5}]) and the distance effect (-0.65 m^{-1} [-0.57 m^{-1}]) were small, although the latter was significantly different from the null hypothesis ($p < .01$) (Figure S2). This suggests that there was still a relation between motion and FC, even after our preprocessing pipeline. As above, DMN intra-connectivity strongly correlated with mean movement.

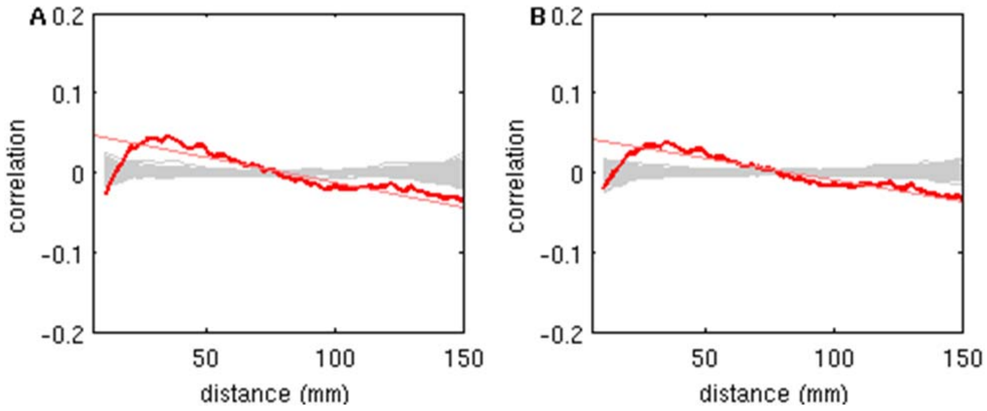

Figure S2. Moving average of correlation between maximum framewise displacement (**A**) or mean framewise displacement (**B**) and functional connectivity against distance between nodes, for the replication dataset. The bold red lines reflect values from actual data, whilst straight red lines are fitted linear functions: gray lines were obtained by permuting movement values for participants.

Correlation Between DMN Connectivity and Motion

A correlation between motion and FC has been found in many studies. Although often considered artifactual, recent work has shown that, for the DMN in particular, weaker connectivity could also be a stable neurobiological trait that predisposes to movement (35). These authors found DMN connectivity to be similar for high- and low-motion scans when these scans were from the same participant, but connectivity to differ when the scans were from distinct participants. This implies that the reduced DMN connectivity associated with movement is not the result of artifacts for the particular scan that contains high movement, but a neurobiological phenotype of certain individuals, shown in scans both when the individual moves much or little.

To further inspect whether the correlation between connectivity and motion may be particularly strong in the DMN in our data, we plotted the correlation values for edges within the DMN, and for all other edges. We found the DMN edges to be more strongly affected by motion than non-DMN edges (Figure S3). Given the evidence by Zeng *et al.*, this residual correlation between DMN and motion may be the result of an inherent trait. As our own dataset contained repeated measurements, each subject was scanned not just at rest but also

during three tasks, we were able to perform a test along the lines of Zeng *et al.* We asked whether subject motion during tasks would predict DMN connectivity in rest, after correction for the motion in rest. If the correlation between DMN connectivity and motion were artifactual, no further information should be contained in the task motion for the rest connectivity. If DMN connectivity were a sign of a neurobiological trait that predisposes to movement, we should find that motion in task predicts rest connectivity. In other words, a subject that moves a lot in the tasks but happens to lie still during rest would still have low DMN connectivity during rest, and vice versa.

We tested whether motion in different scans predicted connectivity during rest using a partial correlation, which measures the degree of association between mean movement during task, x, and DMN connectivity, y, removing the effect of motion during rest, z. That is, we correlated 1) the residuals obtained after regressing x against z with 2) the residuals obtained after regressing y against z. We found a significant partial correlation of −0.19 (p = .03), corroborating the possibility that the correlation between DMN connectivity and motion may be of neurobiological interest, rather than an artifact.

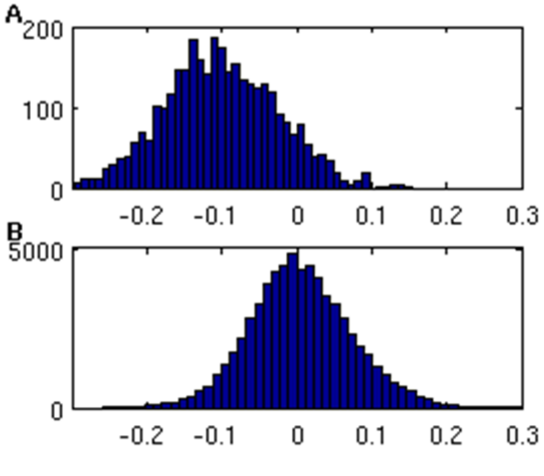

Figure S3. Correlations between mean motion and FC for (**A**) edges in the DMN and (**B**) edges not in the DMN. There was a negative correlation for the DMN, which was not present in the rest of the network.

Parcellation of the Default Mode Network

DMN parcellation comprised 58 regions of interest (36). These regions included nodes in the following brain regions: frontal pole (9); paracingulate gyrus (8); posterior middle temporal gyrus (7); superior lateral occipital cortex (5); posterior cingulate (4); precuneus cortex (4); superior frontal gyrus (4); temporal pole (3); anterior cingulate (2). It also includes singular nodes in the following regions: lateral occipital cortex; angular gyrus; medial frontal cortex; orbitofrontal cortex; left hippocampus; lingual gyrus; middle frontal gyrus; anterior middle temporal gyrus; posterior parahippocampal gyrus; right cerebellar crucible; anterior superior temporal gyrus; posterior fusiform cortex.

Computation of the DMN Connectivity Metric

We defined connectivity between each pair of brain regions for each participant as the Pearson correlation of the voxel-average regional time series. We then thresholded the connectivity matrix for each participant, only keeping the 20% (0.2) strongest connections, in order to remove weak and spurious connections. We defined our measure of DMN intra-connectivity as the fraction of connections between DMN regions that survived the threshold, corrected for the fraction of such connections expected if the DMN were as strongly connected as any part of the brain. More formally, we defined our measure as,

$$M_{\text{DMN}} * \frac{2}{N_{\text{DMN}}(N_{\text{DMN}}-1)} - P_{\text{threshold}}$$

Where M_{DMN} is the number of observed connections between DMN regions, N_{DMN} is the number of DMN regions, and $P_{\text{threshold}} = 0.2$ is the connectivity threshold. Below, we describe a sensitivity analysis that uses a measure that does not depend on a threshold.

Computation of Difference in Connectivity Between Groups

To get an idea of the magnitude of any difference in DMN connectivity found, we computed relative connectivity strength. For each individual in our dataset, we computed the predicted DMN intra-connectivity from our regression model. We computed the mean m_i for each group i of interest, as the average of the predicted values for each individual from that group. We then computed the relative increase from group i over group j as $(m_i - m_j) / m_j$. We took the control males as our baseline group m_j, and female controls, males with ASC and female siblings as the groups m_i.

Robustness Analyses

To examine the robustness of our regression results, we computed additional models with important variations on our original methodology for our replication dataset. We aimed to test whether our original effects, particularly those relating to sex and ASC diagnosis, remained consistent or were due to the configuration of our tests. The analyses are described below, and the results are presented in Table S7. Note that the high intercept shown in this table simply means that the DMN is more strongly connected than randomly chosen brain regions. We highlighted the regressors of prime interest in bold. These are taken to the appropriate baseline, e.g. *Males with ASC – Male controls* refers to the beta associated with males with ASC, when male controls are taken as the baseline.

Exclusion of High-Motion Participants

To ensure results were not driven by a small group of subjects showing high motion, we excluded all participants with maximum FD larger than 2.5 mm, where FD was calculated as the summed absolute values of derivatives of the translational and rotational realignment

estimates, after converting rotational estimates to displacement at 50 mm radius (31). 196 participants were discarded, leaving 784 (80%) participants in the analysis.

Additional Preprocessing: Scrubbing

Although our preprocessing pipeline was designed to remove artifacts caused by motion, we additionally tested whether our results are sensitive to the more radical technique of scrubbing. This technique consists of discarding data from volumes taken during excessive motion. We discarded all data points for all participants for which FD > 0.25, and excluded participants with fewer than 70%, or fewer than 100 data points remaining. This left 751 participants (77%).

Threshold-free Measure of Intra-DMN Connectivity

The measure of DMN connectivity we used in the main text is based on a threshold, aimed to reduce the effect of spurious correlations. To test for sensitivity to this approach, we reran our analysis, quantifying intra-DMN connectivity as the average correlation weight between all the nodes in the DMN, after subtracting the mean connectivity weight across the brain for each participant.

This alternative measure also gave us an alternative to calculating the group effect sizes. We again calculated the percentage decrease/increase of DMN intra-connectivity of ASC males (control females) with respect to control males, and found 17% (21%). This was comparable with the values found in the main text of 16% (27%).

Regressing Out Motion

Although it is clear that in-scanner motion is related to our connectivity measure (see section Motion), we here tested whether the group effect could be explained solely by movement. We therefore included both maximum and mean movement as regressors.

Excluding Studies Previously Reporting DMN Differences

There are a number of studies in the literature that report DMN functional connectivity differences between subjects with ASC and controls. Three of the sites in the ABIDE dataset contain participants analyzed in these studies (UM, Stanford, Olin (15; 16; 22; 23)). To avoid potential for circularity, we repeated our analysis following complete removal of data from these three sites. Note that we had to remove all data from the three sites as we could not establish which of the participants included in ABIDE are those included in the published studies.

Table S7. Results of robustness analyses. Reported are demographics and estimated effect sizes from regression modeling, multiplied by 10^2 to save space. The UCLA study is taken as baseline.

	**Main Text**	**Exclusion of High-Motion Participants** [a]	**With Scrubbing** [a]	**Alternative Measure of DMN Connectivity** [a]	**Regressing Mean and Maximum Movement** [a]	**Excluding Previous DMN Literature** [a]
n Males with ASC	408	301	285	408	408	329
n Male Controls	428	361	345	428	428	344
n Females with ASC	55	40	39	55	55	41
n Female Controls	89	82	82	89	89	66
Intercept	18.56 ***	24.3 ***	24.37 ***	12.57 ***	25.51 ***	18.89 ***
**Males with ASC – Male Controls**	− 3.38 ***	-2.9 **	− 2.64 **	− 2.4 ***	− 2.15 **	− 2.7 **
**Female Controls – Male Controls**	4.2 **	2.91 *	3.83 **	2.68 **	3.44 **	5.13 **
**Females with ASC – Female Controls**	− 5.27 **	− 3.79 '	− 4.59 *	− 3.5 **	− 3.95 *	− 6.2 **
Age	− 0.06	− 0.19 **	− 0.1	− 0.01	− 0.17 **	− 0.09
IQ	0.02	0.00	0.02	0.01	0.01	0.02
UM	0.6	2.44	− 0.65	− 1.98 *	0.59	−
NYU	4.04 **	2.75	− 0.91	2.23 *	1.9	4.12 **

	Main Text	Exclusion of High-Motion Participants [a]	With Scrubbing [a]	Alternative Measure of DMN Connectivity [a]	Regressing Mean and Maximum Movement [a]	Excluding Previous DMN Literature [a]
Yale	7.46 ***	6.24 **	2.03	5.16 ***	5.83 **	7.4 ***
USM	− 1.72	− 1.98	− 5.12 *	− 3.2 **	− 2.04	− 1.46
Stanford	− 2.17	− 1.65	− 7.29 **	− 1.67	− 3.84 '	−
SJH	0.7	− 0.26	− 3.4	2.18 '	− 0.45	− 1.46
SDSU	3.87	2.55	− 1.46	2.27	2.04	3.82
Pitt	1.34	1.01	− 1.84	0.1	0.82	1.51
Olin	0.79	2.98	− 2.25	− 0.86	2.55	−
OHSU	− 12.63 ***	− 14.48 ***	−	− 3.29 *	− 14.78 ***	3.82
MPG	− 3.01	− 3.3	− 8.49 **	− 0.5	− 2.85	1.51
KUL	6.92 ***	6.49 **	2.49	3.2 **	5.48 **	7.11 ***
KKI	− 4.28 *	− 5.16 *	− 8.01 **	− 1.24	− 4.3 *	− 4.34 *
Caltech	− 1.58	− 1.62	− 4.59	− 0.1	− 2.12	− 1.06
Max Motion	−	−	−	−	− 0.27	
Mean Motion	−	−	−	−	− 10.87 ***	

[a] See Robustness Analyses section, above.
*** $p < .001$.
** $p < .01$.
* $p < .05$.
’ $p < .1$.

Different Age Groups

We investigated whether the effects were present throughout the lifespan. We repeated our robust regression analysis, for children (aged below 12), adolescent (aged 12-18) and adults (aged above 18). We reproduced results for children and adolescents, but not adults (Table S8).

Table S8. Results of analyses per age group. Reported are demographics and estimated effect sizes from regression modeling, multiplied by 10^2 to save space. The UCLA study is taken as baseline.

	**Main Text**	**Children**	**Adolescents**	**Adults**
n Males with ASC	443	118	227	98
n Male Controls	448	111	215	122
n Females with ASC	71	22	39	10
n Female Controls	109	27	66	16
Intercept	19.27 ***	9.35	13.76 *	21.46 *
**Males with ASC – Male Controls**	− 3.53 ***	− 3.69 *	− 3.46 **	− 2.71 '
**Female Controls – Male Controls**	4.96 ***	6.29 *	5.34 **	1.94
**Females with ASC – Female Controls**	− 7.00 ***	− 8.42 **	− 5.68 *	− 7.81 '
Age	− 0.05	0.95 '	0.05	− 0.15
IQ	0.02	− 0.01	0.07 '	0.02
UM	0.48	− 1.48	0.33	–
NYU	3.96 **	6.19 **	4.56 *	2.84
Yale	7.37 ***	6.21 *	8.52 **	–
USM	− 1.83	− 1.3	− 2.15	− 2.89
Stanford	− 2.12	1.33	− 5.74	–
SJH	0.65	–	− 1.1	− 0.05
SDSU	3.65	− 6.63	2.54	–
Pitt	1.29	3.41	1.56	− 1.29
Olin	0.78	− 7.45	2.02	− 3.54
OHSU	− 12.59 ***	− 7.89 *	− 22.47 ***	–
MPG	− 3.12	− 1.11	− 3.46	− 2.27
KUL	6.82 ***	–	5.55 *	5.48
KKI	− 4.31 *	− 1.94	− 1.35	–
Caltech	− 1.75	–	− 6.99	− 1.07
Sibling	2.5	–	0.54	–

*** $p < .001$.
** $p < .01$.
* $p < .05$.
’ $p < .1$.

Positive Control: Depression

We performed two robust regression analyses to test the specificity of DMN intra-connectivity to ASC using the MR-IMPACT study data, combined with the pooled CFSA and ABIDE data. Firstly, we included age, sex, study and diagnosis as predictors. Secondly, we tested for specific effects in one of the sexes, by including an interaction effect between diagnosis and sex. Table S9 shows no significant effects of depression were found.

Table S9. Results of analyses including a positive control. Reported are demographics and estimated effect sizes from regression modeling, multiplied by 10^2 to save space. Study effects are compared to UCLA as a baseline.

	**Diagnosis Effect**	**Per Group**
n Males with ASC	443	443
n Male Controls	454	454
n Females with ASC	71	71
n Female Controls	127	127
n Males with Depression	17	17
n Females with Depression	46	46
**ASC - Control**	− 4.19 ***	−
**Depression - Control**	0.59	−
**Females - Males**	3.53 ***	−
**Females with Depression – Female Controls**	−	− 0.04
**Males with Depression – Male Controls**	−	2.45
Age	− 0.04	− 0.04
UM	0.66	0.5
NYU	4.11 **	4.02 **
Yale	7.31 ***	7.26 ***
USM	− 1.77	− 1.89
Stanford	− 1.97	− 1.95
SJH	0.77	0.7
SDSU	3.94	3.65
Pitt	1.36	1.3
Olin	0.94	0.89
OHSU	− 12.38 ***	− 12.41 ***
MPG	− 3.18	− 3.14
KUL	6.85 ***	6.81 ***
KKI	− 4.22 *	− 4.25 *
Caltech	− 1.72	− 1.82
Sibling	2.6	2.56
MR-Impact	2.73	1.8

*** $p < .001$.
** $p < .01$.
* $p < .05$.

Further Analysis of Behavioral Data

We examined whether DMN intra-connectivity correlated with performance in the mentalizing and control task (making gender judgments), quantified as the percentage of incorrect responses. We employed robust regression with performance as the outcome variable, and DMN intra-connectivity as the predictor of interest. Other predictors included age, IQ, and the group of the participant (males with ASC, females with ASC, male siblings, female siblings, male controls and female controls). Thus, any correlation found demonstrates an effect beyond the effect of group. We repeated this analysis stratifying for sex. There were two clear outliers on the mentalizing task, showing error rates above 50%; we repeated the male-only analysis after discarding these outliers. Finally, we repeated the full analysis, including maximum and mean movement in the analysis.

Overall, we found a strong effect of DMN intra-connectivity and IQ for the mentalizing task, for both of the sexes (Table S10). No significant effects were found for the control task (Table S11). Motion did not correlate with performance in either task.

Table S10. Results of analyses on performance on a mentalizing task. Reported are demographics and estimated effect sizes from regression modeling, multiplied by 10^2 to save space. Group effects are compared to males with ASC, to females with ASC for the female-only analysis, and to the male group for the three group-stratified analyses.

	Full Analysis	Females	Males	Full, Including Motion	ASC	Siblings	Controls
n Males with ASC	35	–	35	35	35	-	-
n Male Controls	20	–	20	20	-	20	-
n Males Siblings	13	–	13	13	-	-	13
n Females with ASC	16	16	–	16	16	-	-
n Female Controls	20	20	–	20	-	20	-
n Females Siblings	30	30	–	30	-	-	30
Intercept	41.9 ***	44.95 **	42.45 ***	44.63 ***	57.29 **	19.53	45.55 **
**DMN Intra-connectivity**	− 22.59 **	− 21.1 *	− 23.74 *	− 23.75 ***	-39.92 **	-8.78	-18.16
Age	− 0.15	− 0.03	− 0.39	− 0.19	-0.83	0.59	0.01
IQ	− 0.18 **	− 0.23 *	− 0.15 '	− 0.19 **	-0.19 '	-0.13	-0.25 '
Male Controls	− 2.74	–	− 2.6	− 2.51	-	-	-
Male Siblings	− 1.05	–	− 0.99	− 1.02	-	-	-
Females with ASC	− 0.18	–	–	− 0.44	-0.33	-	-
Female Controls	− 1.03	− 0.42	–	− 0.39	-	0.66	-
Female Siblings	− 1.41	− 0.73	–	− 1.43	-	-	-0.42
Mean Motion	–	–	–	− 7.92	-	-	-
Max Motion	–	–	–	0.62	-	-	-

*** $p < .001$.
** $p < .01$.
* $p < .05$.
’ $p < .1$.

Table S11. Results of analyses on performance on a control task. Reported are demographics and estimated effect sizes from regression modeling, multiplied by 10^2 to save space. Group effects are compared to males with ASC, to females with ASC for the female-only analysis, and to the male group for the three group-stratified analyses.

	Full Analysis	Females	Males	Full, Including Motion	ASC	Siblings	Controls
n Males with ASC	35	-	35	35	35	-	-
n Male Controls	20	-	20	20	-	20	-
n Males Siblings	13	-	13	13	-	-	13
n Females with ASC	16	16	-	16	16	-	-
n Female Controls	20	20	-	20	-	20	-
n Females Siblings	30	30	-	30	-	-	30
Intercept	12.29 **	13.31 *	10.44 '	13.29 **	20.74 *	4.81	9.49
**DMN Intra-connectivity**	1.12	1.86	1.33	1.12	-8.5	5.67	4.68
Age	-0.18	-0.11	-0.28	-0.19	-0.57	0.19	-0.11
IQ	-0.05 '	-0.09 *	-0.02	-0.05 '	-0.06	-0.05	-0.05
Male Controls	-0.84	-	-0.92	-0.94	-	-	-
Male Siblings	-1.62	-	-1.7	-1.68	-	-	-
Females with ASC	-2.13 '	-	-	-2.48 *	-1.94	-	-
Female Controls	-1.59	1.25	-	-1.52	-	-0.8	-
Female Siblings	-1.12	1.78	-	-1.26	-	-	0.35
Mean Motion	-	-	-	-3.1	-	-	-
Max Motion	-	-	-	0.23	-	-	-

** $p < .01$.
* $p < .05$.
’ $p < .1$.

Supplemental References

1. Spencer MD, Holt RJ, Chura LR, Suckling J, Calder AJ, Bullmore ET, Baron-Cohen S (2011): A novel functional brain imaging endophenotype of autism: the neural response to facial expression of emotion. *Transl Psychiatry*. 1: e19.

2. Spencer MD, Holt RJ, Chura LR, Calder AJ, Suckling J, Bullmore ET, Baron-Cohen S (2012): Atypical activation during the Embedded Figures Task as a functional magnetic resonance imaging endophenotype of autism. *Brain*. 135: 3469–3480.

3. Spencer MD, Chura LR, Holt RJ, Suckling J, Calder AJ, Bullmore ET, Baron-Cohen S (2012): Failure to deactivate the default mode network indicates a possible endophenotype of autism. *Mol Autism*. 3: 1–9.

4. Floris DL, Chura LR, Holt RJ, Suckling J, Bullmore ET, Baron-Cohen S, Spencer MD (2013): Psychological correlates of handedness and corpus callosum asymmetry in autism: the left hemisphere dysfunction theory revisited. *J Autism Dev Disord*. 43: 1758–72.

5. Holt RJ, Chura LR, Lai M-C, Suckling J, von dem Hagen E, Calder AJ, *et al.* (2014): "Reading the Mind in the Eyes": an fMRI study of adolescents with autism and their siblings. *Psychol Med*. 44: 3215–27.

6. Moseley RL, Ypma RJF, Holt RJ, Floris D, Chura LR, Spencer MD, *et al.* (2015): Whole-brain functional hypoconnectivity as an endophenotype of autism in adolescents. *NeuroImage Clin*. 9: 140–152.

7. Lisiecka DM, Holt R, Tait R, Ford M, Lai M-C, Chura LR, *et al.* (2015): Developmental white matter microstructure in autism phenotype and corresponding endophenotype during adolescence. *Transl Psychiatry*. 5: e529.

8. Lord C, Risi S, Lambrecht L, Cook EH, Leventhal BL, Dilavore PC, *et al.* (2000): The Autism Diagnostic Observation Schedule-Generic: A standard measure of social and communication deficits associated with the spectrum of autism. *J Autism Dev Disord*. 30: 205–223.

9. Le Couteur A, Lord C, Rutter M (2003): *The autism diagnostic interview-revised (ADI-R)*. Los Angeles, CA: Western Psychological Services.

10. Baron-Cohen S, Wheelwright S, Skinner R, Martin J, Clubley E (2001): The Autism-Spectrum Quotient (AQ): Evidence from Asperger Syndrome/High-Functioning Autism, Males and Females, Scientists and Mathematicians. *J Autism Dev Disord*. 31: 5–17.

11. Rutter M, Bailey A, Lord C (2003): *Social Communication Questionnaire. Los Angeles, CA*. Los Angeles, CA: Western Psychological Services.

12. Di Martino A, Yan C-G, Li Q, Denio E, Castellanos FX, Alaerts K, *et al.* (2014): The autism brain imaging data exchange: towards a large-scale evaluation of the intrinsic brain architecture in autism. *Mol Psychiatry*. 19: 659–67.

13. Rudie JD, Hernandez LM, Brown JA, Beck-Pancer D, Colich NL, Gorrindo P, *et al.* (2012): Autism-Associated Promoter Variant in MET Impacts Functional and Structural Brain Networks. *Neuron*. 75: 904–915.

14. Rudie JD, Shehzad Z, Hernandez LM, Colich NL, Bookheimer SY, Iacoboni M, Dapretto M (2012): Reduced functional integration and segregation of distributed neural systems underlying social and emotional information processing in Autism spectrum disorders. *Cereb Cortex*. 22: 1025–1037.

15. Monk CS, Peltier SJ, Wiggins JL, Weng SJ, Carrasco M, Risi S, Lord C (2009): Abnormalities of intrinsic functional connectivity in autism spectrum disorders,. *Neuroimage*. 47: 764–772.

16. Weng S-J, Wiggins JL, Peltier SJ, Carrasco M, Risi S, Lord C, Monk CS (2010): Alterations of resting state functional connectivity in the default network in adolescents with autism spectrum disorders. *Brain Res*. 1313: 202–214.

17. Wiggins JL, Peltier SJ, Ashinoff S, Weng S-J, Carrasco M, Welsh RC, *et al.* (2011): Using a self-organizing map algorithm to detect age-related changes in functional connectivity during rest in autism spectrum disorders. *Brain Res*. 1380: 187–197.
18. Di Martino A, Zuo XN, Kelly C, Grzadzinski R, Mennes M, Schvarcz A, *et al.* (2013): Shared and distinct intrinsic functional network centrality in autism and attention-deficit/hyperactivity disorder. *Biol Psychiatry*. 74: 623–632.
19. Anderson JS, Nielsen JA, Froehlich AL, Dubray MB, Druzgal TJ, Cariello AN, *et al.* (2011): Functional connectivity magnetic resonance imaging classification of autism. *Brain*. (Vol. 134), pp 3739–3751.
20. Anderson JS, Druzgal TJ, Froehlich A, Dubray MB, Lange N, Alexander AL, *et al.* (2011): Decreased interhemispheric functional connectivity in autism. *Cereb Cortex*. 21: 1134–1146.
21. Uddin LQ, Supekar K, Lynch CJ, Khouzam A, Phillips J, Feinstein C, *et al.* (2013): Salience network-based classification and prediction of symptom severity in children with autism. *JAMA psychiatry*. 70: 869–79.
22. Lynch CJ, Uddin LQ, Supekar K, Khouzam A, Phillips J, Menon V (2013): Default mode network in childhood autism: Posteromedial cortex heterogeneity and relationship with social deficits. *Biol Psychiatry*. 74: 212–219.
23. Assaf M, Jagannathan K, Calhoun VD, Miller L, Stevens MC, Sahl R, *et al.* (2010): Abnormal functional connectivity of default mode sub-networks in autism spectrum disorder patients. *Neuroimage*. 53: 247–256.
24. Tyszka JM, Kennedy DP, Paul LK, Adolphs R (2014): Largely typical patterns of resting-state functional connectivity in high-functioning adults with autism. *Cereb Cortex*. 24: 1894–1905.
25. Hagan CC, Graham JM, Widmer B, Holt RJ, Ooi C, van Nieuwenhuizen AO, *et al.* (2013): Magnetic resonance imaging of a randomized controlled trial investigating predictors of recovery following psychological treatment in adolescents with moderate to severe unipolar depression: study protocol for Magnetic Resonance-Improving Mood with Psycho. *BMC Psychiatry*. 13: 247.
26. American Psychiatric Association (2000): *Diagnostic and Statistical Manual of Mental Disorders*, Fourth. Washington, DC: American Psychiatric Association.
27. Power JD, Mitra A, Laumann TO, Snyder AZ, Schlaggar BL, Petersen SE (2014): Methods to detect, characterize, and remove motion artifact in resting state fMRI. *Neuroimage*. 84: 320–41.
28. Power JD, Schlaggar BL, Petersen SE (2015): Recent progress and outstanding issues in motion correction in resting state fMRI. *Neuroimage*. 105: 536–551.
29. Satterthwaite TD, Wolf DH, Loughead J, Ruparel K, Elliott MA, Hakonarson H, *et al.* (2012): Impact of in-scanner head motion on multiple measures of functional connectivity: Relevance for studies of neurodevelopment in youth. *Neuroimage*. 60: 623–632.
30. van Dijk KRA, Sabuncu MR, Buckner RL (2012): The influence of head motion on intrinsic functional connectivity MRI. *Neuroimage*. 59: 431–438.
31. Power JD, Barnes KA, Snyder AZ, Schlaggar BL, Petersen SE (2012): Spurious but systematic correlations in functional connectivity MRI networks arise from subject motion. *Neuroimage*. 59: 2142–2154.
32. Jenkinson M, Bannister P, Brady M, Smith S (2002): Improved optimization for the robust and accurate linear registration and motion correction of brain images. *Neuroimage*. 17: 825–841.
33. Power JD, Barnes KA, Snyder AZ, Schlaggar BL, Petersen SE (2013): Steps toward optimizing motion artifact removal in functional connectivity MRI; a reply to Carp. *Neuroimage*. 76: 439–441.
34. Patel AX, Kundu P, Rubinov M, Jones PS, Vértes PE, Ersche KD, *et al.* (2014): A wavelet method for modeling and despiking motion artifacts from resting-state fMRI time series. *Neuroimage*. 95: 287–304.

35. Zeng L-L, Wang D, Fox MD, Sabuncu M, Hu D, Ge M, *et al.* (2014): Neurobiological basis of head motion in brain imaging. *Proc Natl Acad Sci U S A*. 111: 6058–62.

36. Power JD, Cohen AL, Nelson SM, Wig GS, Barnes KA, Church J a, *et al.* (2011): Functional network organization of the human brain. *Neuron*. 72: 665–78.
